# Virulence plasmids in edema disease: Insights from whole-genome analysis of porcine O139:H1 Shiga toxin-producing *Escherichia coli* (STEC) strains

**DOI:** 10.3389/fcimb.2025.1528408

**Published:** 2025-03-20

**Authors:** Ali Nemati, Federica Gigliucci, Stefano Morabito, Mahdi Askari Badouei

**Affiliations:** ^1^ European Union Reference Laboratory (EURL) for Escherichia coli including Shiga toxin-producing E. coli (STEC), Department of Food Safety, Nutrition and Veterinary Public Health, Istituto Superiore di Sanità, Rome, Italy; ^2^ Department of Pathobiology, Faculty of Veterinary Medicine, Ferdowsi University of Mashhad, Mashhad, Iran

**Keywords:** plasmid, O139:H1, Shiga toxin-producing *Escherichia coli*, STEC, Edema disease

## Abstract

This study investigates the plasmid sequences of porcine O139:H1 Shiga toxin-producing *Escherichia coli* (STEC) responsible for Edema Disease (ED). Whole-genome analysis reveals significant similarities between these strains and known plasmids, notably pW1316-2, which harbors key virulence genes like hemolysin (*hlyA*, *hlyB*) and adhesion factors (*aidA-I*, *faeE*). These genes contribute to the cytotoxicity and host colonization associated with ED. Additionally, similarities to plasmids from *Shigella flexneri* 2a highlight potential associations in virulence gene regulation, particularly via the *Hha-H-NS* complex. The identification of sequences resembling plasmid pB71 raises serious concerns about the emergence of highly pathogenic strains, as it includes tetracycline resistance genes (*tetA*, *tetC*, *tetR*). This research emphasizes the role of plasmid-like sequences in ED pathogenesis, indicating important implications for swine industry management and public health.

## Background

Edema Disease (ED) is a sudden and severe form of toxemia caused by Shiga toxin-producing *Escherichia coli* (STEC) strains (Moxley, 2000). These isolates produce F18 fimbrial adhesins, α-hemolysin, and Shiga toxin 2e (*stx2e*), primarily affecting healthy, rapidly growing nursery pigs (Fairbrother and Nadeau, 2019). Following adhesion of STEC to the intestinal mucosa mediated by F18, *stx2e* enters the bloodstream, leading to vascular damage in various organs, including the brain and gastrointestinal tract (Marques et al., 1987). ED poses a significant economic burden on the pig industry, and its treatment is often ineffective due to the disease’s sudden onset and rapid progression (Gale and Velazquez, 2020).

The prevalent STEC serogroups associated with ED include O138, O139, and O141, with a notable presence of serotypes O139:K82:H1, O141:K85:H4, and O138:K81:NM (Perrat et al., 2022). Key virulence factors, beside the F18 fimbriae, contributing to ED development include *Stx2e* and α-hemolysin (Gale and Velazquez, 2020). Their coding genes are typically harbored on plasmids, except for the *stx2e* gene, which is commonly integrated into the chromosome within a prophage (Denamur et al., 2021; Gigliucci et al., 2021). The initial colonization of the porcine intestine by STEC causing ED is primarily mediated by F18 (F18ab or F18ac) and F4 (K88) adhesins (DebRoy et al., 2009). Additionally, bacterial AIDA (adhesin involved in diffuse adherence) also contributes to the initial phases of the pathogenic process (Gale and Velazquez, 2020). These adhesins are also encoded by genes described in plasmids of Enterobacterales members other than *E. coli*, particularly *Shigella* spp. and *Salmonella enterica*, indicating a potential mechanism of acquisition of these adhesins by STEC causing ED from these species (Keshmiri et al., 2022; Badouei et al., 2023). Horizontal transfer of virulence plasmids among STEC pathotypes is described and could be a mean to enhance the pathogenicity of ED strains and eventually leading to the emergence of hybrid pathotypes (Cointe et al., 2018; Tomeh et al., 2024). Characterizing these strains is therefore crucial for assessing their virulence potential, facilitating the development of detection methods, and understanding their evolution, to unravel potential implications for public health (Nemati et al., 2024).

Plasmid-encoded genes of porcine O139 STEC strains thus can influence various stages of ED pathophysiology, including those related with adhesion, invasion, colonization, and modulation of host immune responses (Fairbrother and Nadeau, 2019). Additionally, food contamination with STEC may pose significant concerns for food safety and public health (Tseng et al., 2014). In this study, we conducted a whole-genome sequencing-based study on a collection of Italian O139 STEC strains isolated from pigs with ED to investigate the structure of their plasmid-like sequences and to elucidate the possible ways such a plasmid was acquired by ED-associated STEC as well as the possibility to identify new relevant virulence factors carried on these sequences.

## Methods

### Bacterial strains

We analyzed a collection of 53 STEC O139:H1 strains isolated from pigs affected by ED in Italy, maintained in the National Reference Laboratory for *E. coli* collections at the Istituto Superiore di Sanità. Additionally, we included in this study 83 more STEC O139:H1 genomes retrieved from the GenBank and the European Nucleotide Archive (ENA) databases, from strains isolated from pigs or other sources (**Supplementary Tables 1**, **2**).

### Whole-genome sequencing

To conduct whole genome sequencing (WGS), total DNA was extracted from a 2 mL overnight TSB culture of each strain grown at 37°C using the GRS Genomic DNA Kit Bacteria (GRISP Research Solutions, Porto, Portugal). The majority of sequences were generated using Ion Torrent sequencing technology (Thermo Fisher Scientific, MA, USA). Sequencing libraries of approximately 400 bp were prepared from 100 ng of total DNA using the NEBNext Fast DNA Fragmentation & Library Prep Set for Ion Torrent (New England BioLabs, MA, USA). These libraries were then processed through emulsion PCR and enrichment on the Ion OneTouch 2 System, followed by sequencing on an Ion Torrent S5 platform (Thermo Fisher Scientific, MA, USA) using the ION 520/530 KIT-OT2 (Thermo Fisher Scientific, MA, USA) according to the manufacturer’s instructions. All genomic sequences are accessible at the GenBank (BioProject: PRJNA1152229).

### Bioinformatic analyses

Most of the bioinformatic analyses to characterize the genomes were carried out using the tools available on the Galaxy public server ARIES (Istituto Superiore di Sanità, https://www.iss.it/site/aries) (Knijn et al., 2020).

### Characterization of STEC strains

Single-end reads from the Ion Torrent S5 platform were assembled using SPADES version 3.12.0 with default parameters (Bankevich et al., 2012), and filtered using the Filter SPAdes repeats tool (https://github.com/phac-nml/galaxy_tools) with default settings to eliminate repeated contigs or those <1,000 bases in length. Paired-end reads were trimmed, filtered using the Extended Randomized Numerical alignEr–filter (Del Fabbro et al., 2013), and *de novo* assembled using SPAdes version 3.10.0 (Bankevich et al., 2012).

Multilocus sequence typing was performed using the MentaLiST tool version 0.2.3 (Feijao et al., 2018), following the scheme developed by Wirth et al (Wirth et al., 2006). The assembled contigs were analyzed using BLAST (http://blast.ncbi.nlm.nih.gov/Blast.cgi) and the blastn algorithm version 2.7.1 using different databases. Serotyping was determined by aligning the contigs with reference sequences for the O and H antigen genes (Joensen et al., 2015). Subtyping of *stx* was performed using the Shiga toxin typer tool v2.0 (https://github.com/aknijn/shigatoxin-galaxy), which conducts an optimized blastn search against the sequence database of *stx* subtypes developed by the Statens Serum Institut (https://bitbucket.org/genomicepidemiology/virulencefinder_db/src/master/stx.fsa).

### Plasmid identification

BLAST + blastn was also used to detect the presence of plasmid-related sequences and their replicon types within the whole genome of the O139:H1 strains using the PlasmidFinder and PLSDB tools, with a minimum identity of 95% and minimum coverage of 60%. Additionally, we retrieved the most closely related plasmid sequences available online from the National Center for Biotechnology Information (NCBI) nucleotide collection database (accessed June 1, 2024). The focus of this analysis was on determining the similarity of these identified sequences to known reference plasmid sequences.

### Identification of resistance genes, insertion sequence, and virulence factors

The identification of resistance genes was conducted by submitting the complete reference plasmid nucleotide sequence to the ResFinder web server with default parameters, which required a minimum identity of 90% and minimum coverage of 60% (http://genepi.food.dtu.dk/resfinder) (Zankari et al., 2012). Similarly, the reference plasmid was submitted to the VFDB web server via the BLAST sequence-similarity (setB), also with default parameters, to identify virulence factors (http://www.mgc.ac.cn/VFs/search_VFs.htm) (Liu et al., 2022).

### Plasmids comparison

The Prokka tool (Galaxy Version 1.14.5) (Seemann, 2014) was used to perform functional annotation on the assembled sequences of the O139:H1 strains and reference plasmids, utilizing the *E. coli*-specific gene database and default parameters. Additionally, the Blast Ring Image Generator (BRIG) software v0.95 (Alikhan et al., 2011) was used with default parameters to compare and visually represent these plasmid-like sequences in relation to reference plasmids, highlighting regions of similarity and potential functional significance.

### Virulence genes associations

The associations and clustering between the virulence genes identified on the reference plasmids were assessed using the STRING database (https://string-db.org/). The analyses were carried out following the updated instructions (The STRING database in 2023) (Szklarczyk et al., 2023).

### Phylogenomics analysis

We performed the analysis using core genome multilocus sequence typing (cgMLST) with the chewBBACA tool and the INNUENDO project’s scheme, available on the Galaxy public server ARIES, which includes 2,360 loci (Llarena et al., 2018; Silva et al., 2018). Pairwise comparisons were deemed reliable when more than 80% of loci for each sample were assigned to an allele. The distances between strains were calculated by comparing allelic profiles pairwise, using the chewTree tool on the ARIES webserver. For each sample pair, alleles that were missing, partially identified, or incorrectly assigned to any locus were excluded. The resulting dendrogram was visualized using Newick Display on Galaxy Version 1.6 (Junier and Zdobnov, 2010).

## Results

### Genomic characterization of O139:H1 STEC strains

Among the genomes of the 53 Italian strains examined, 50 were classified as ST1, and 3 as ST955. All strains were identified as O139:H1 serotype, with all the 53 strains carrying the *stx2* gene only, specifically subtype *stx2e*.

Among the additional 83 genomes retrieved from the public domain, 79 were classified as ST1, two as ST955, one as ST10859, and one as ST114. Of these, 79 strains carried the *stx2* gene, subtype *stx2e*, while the *stx2* subtypes in four isolates were not identified (**Supplementary Tables 1**, **2**).

### Virulence plasmids

**Figure 1:**
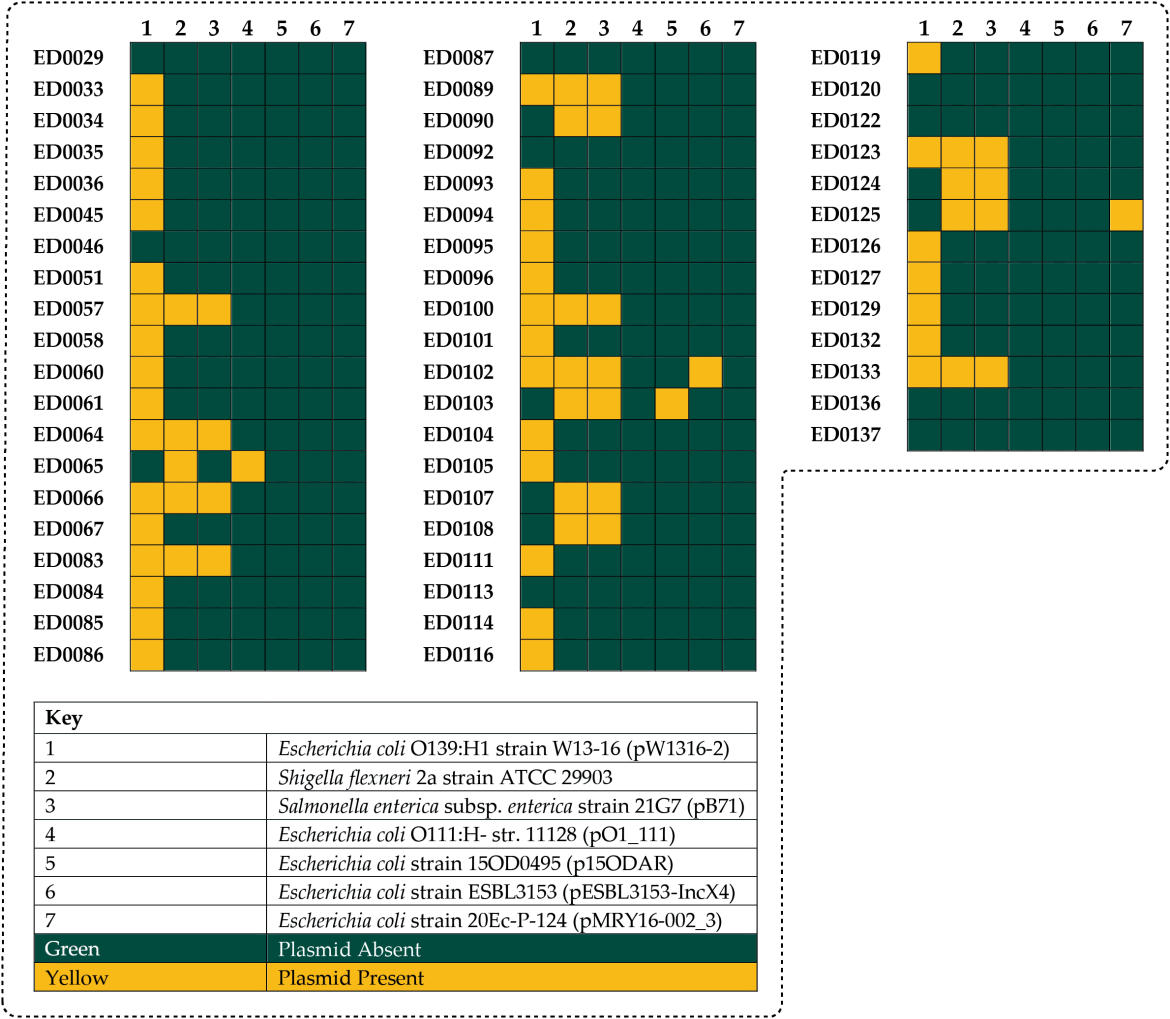
The identified reference plasmids in the genomes of the 53 Italian STEC strains which included: pW1316-2 (37/53, 69.8%), *Shigella flexneri* 2a (16/53, 30.1%), pB71 (15/53, 28.3%), pO111_1 (1/53, 1.8%), p15ODAR (1/53, 1.8%), pESBL3153-IncX4 (1/53, 1.8%), and pMRY16-002_3 (1/53, 1.8%).

Three different reference plasmids were identified in the genome of studied isolates based on the similarity between plasmid-like sequences in the O139:H1 strains and the identified reference plasmid sequences according to the PLSDB (**Figure 1**): *Escherichia coli* O139:H1 strain W13-16 plasmid pW1316-2 (Accession number: NZ_CP080237.1), *Shigella flexneri* 2a strain ATCC 29903 (Accession number: CP026790.1), and *Salmonella enterica* subsp. *enterica* serovar Typhimurium strain 21G7 isolate B71 plasmid pB71 (Accession number: NZ_KP899806.1). Moreover, some genome returned hits against *Escherichia coli* O111:H- str. 11128 plasmid pO111_1 (Accession number: NC_013365.1), *Escherichia coli* strain 15OD0495 plasmid p15ODAR (Accession number: NZ_MG904995.1), *Escherichia coli* strain ESBL3153 plasmid pESBL3153-IncX4 (Accession number: NZ_MW390521.1), and *Escherichia coli* strain 20Ec-P-124 plasmid pMRY16-002_3 (Accession number: NZ_AP017613.1) plasmids (**Figure 1**).

**Figure 2:**
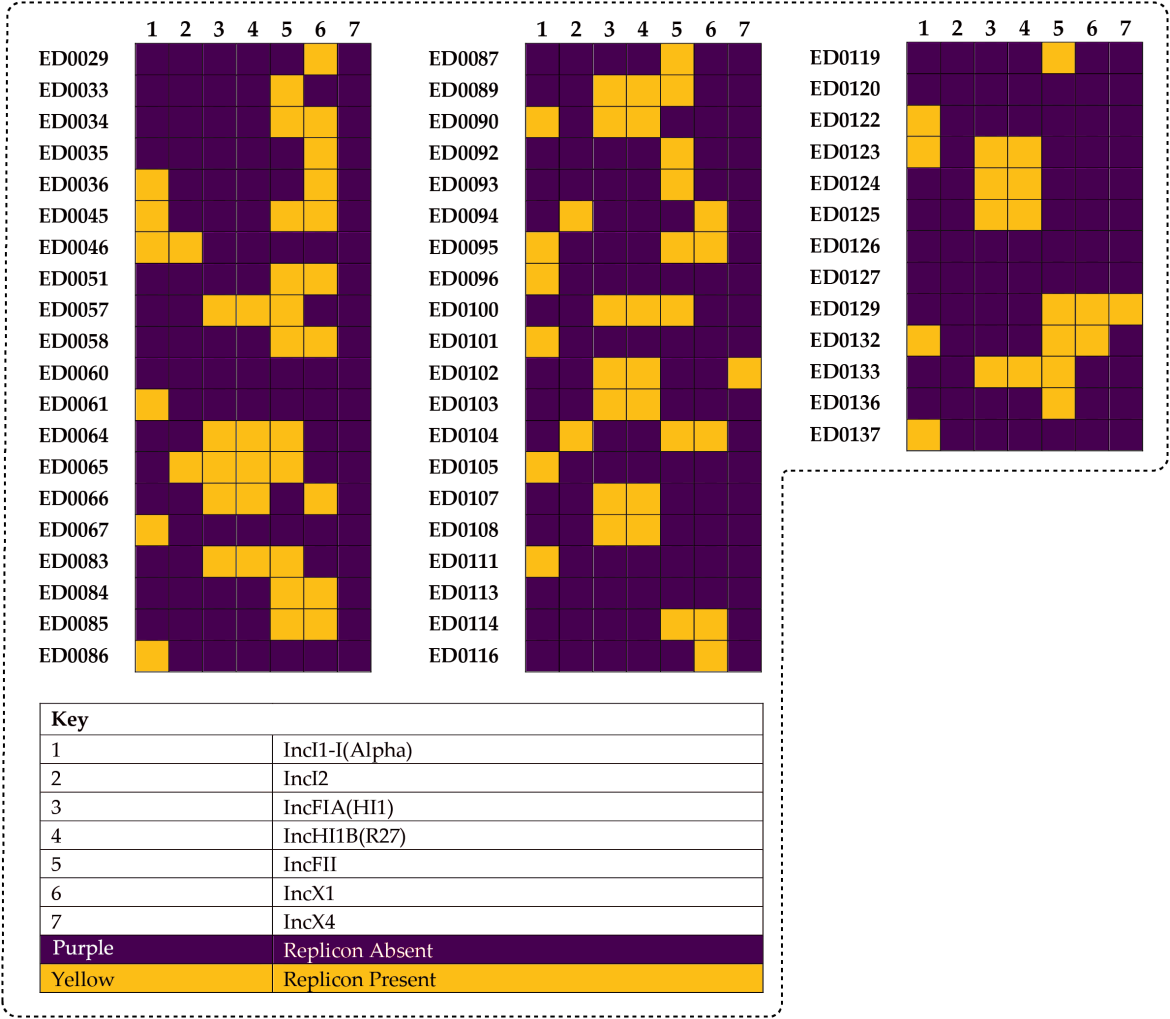
Different plasmid replicon types detected among the identified plasmid-related sequences in the genomes of the 53 Italian STEC strains which included: IncI1-I(Alpha) (16/53, 30.1%), IncI2 (4/53, 7.5%), IncFIA(HI1) (16/53, 30.1%), IncHI1B(R27) (16/53, 30.1%), IncFII (24/53, 45.2%), IncX1 (17/53, 32.0%), and IncX4 (2/53, 3.7%).

**Figure 3:**
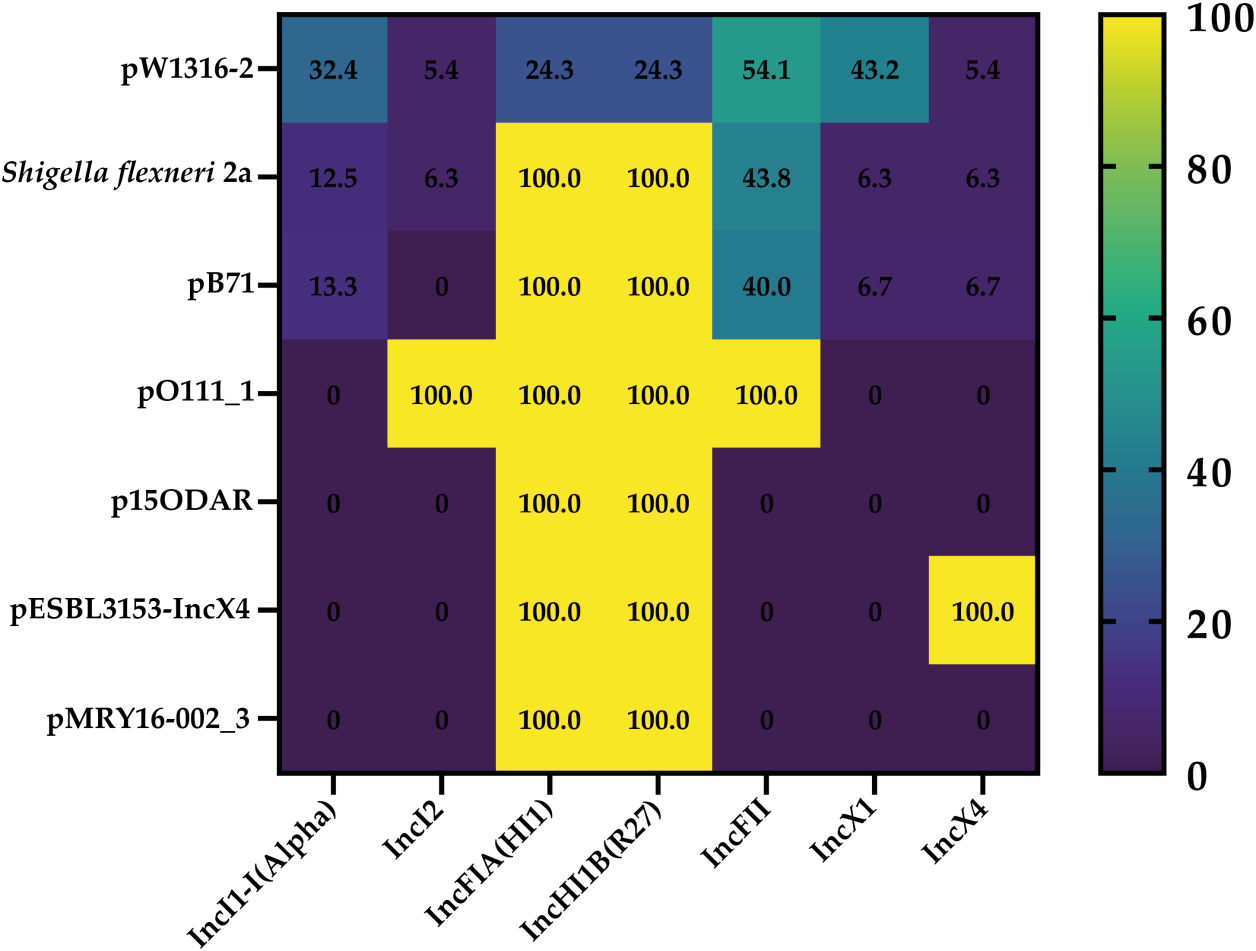
Heatmap generated according to the co-occurrence between identified reference plasmids and different detected replicon types among the genomes of the 53 Italian STEC strains.

We identified seven different replicon types among the plasmid-related sequences in the O139:H1 genomes based on the PlasmidFinder which included: IncI1-I(Alpha), IncI2, IncFIA(HI1), IncHI1B(R27), IncFII, IncX1, IncX4 (**Figures 2**, **3**).

As far as the additional genomes included in the study are concerned, the plasmids *Escherichia coli* O139:H1 strain W13-16 plasmid pW1316-2 (Accession number: NZ_CP080237.1), *Shigella flexneri* 2a strain ATCC 29903 (Accession number: CP026790.1), and *Salmonella enterica* subsp. *enterica* serovar Kentucky plasmid pCS0010A_9 (Accession number: NC_019104.1) were the most frequently identified reference plasmids to which the genomes demonstrated similarity. Moreover, the replicon types IncFII, IncX1, IncI1-I(Alpha), and IncHI1B(R27) were the most frequently detected replicon sequences (**Supplementary Tables 1**, **2**).

### Plasmid-encoded genes

Overall, the most frequent plasmid signatures identified in all strains (O139:H1 STEC isolates of our and other studies), were to pW1316-2, *Shigella flexneri* 2a, and pB71 plasmids.

**Figure 4:**
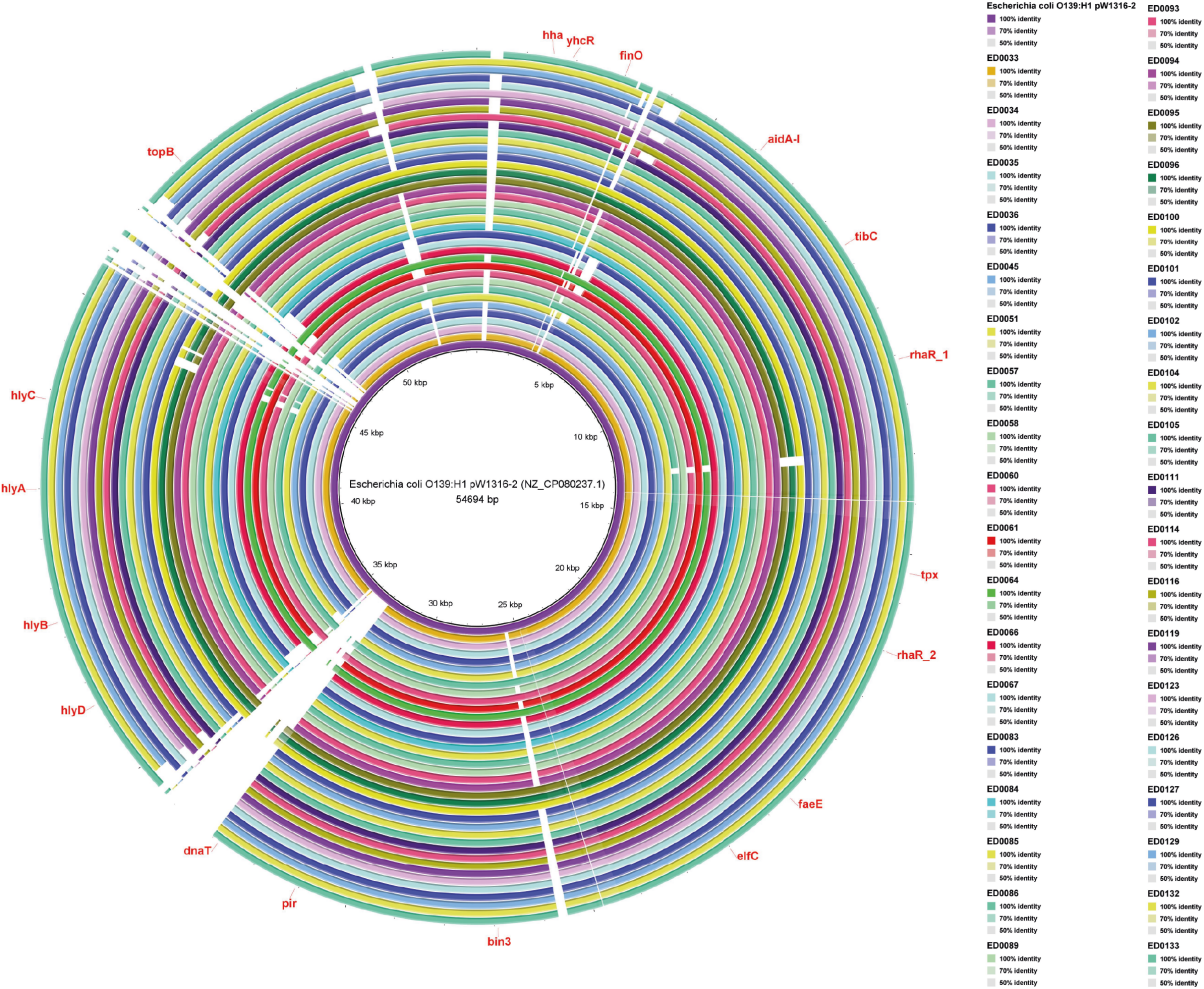
Whole-genome comparison of Blast Ring Image Generated for *Escherichia coli* O139:H1 pW1316-2 (NZ_CP080237.1) plasmid in O139:H1 STEC strains isolated from pigs affected by Edema disease in Italy.

Our analysis using BRIG software revealed the presence of *hha, yhcR, finO, aidA-I, tibC, rhaR_1, tpx, rhaR_2, faeE, elfC, bin3, pir, dnaT, hlyD, hlyB, hlyA, hlyC*, and *topB* genes on the pW1316-2 plasmid (**Figure 4**).

**Figure 5:**
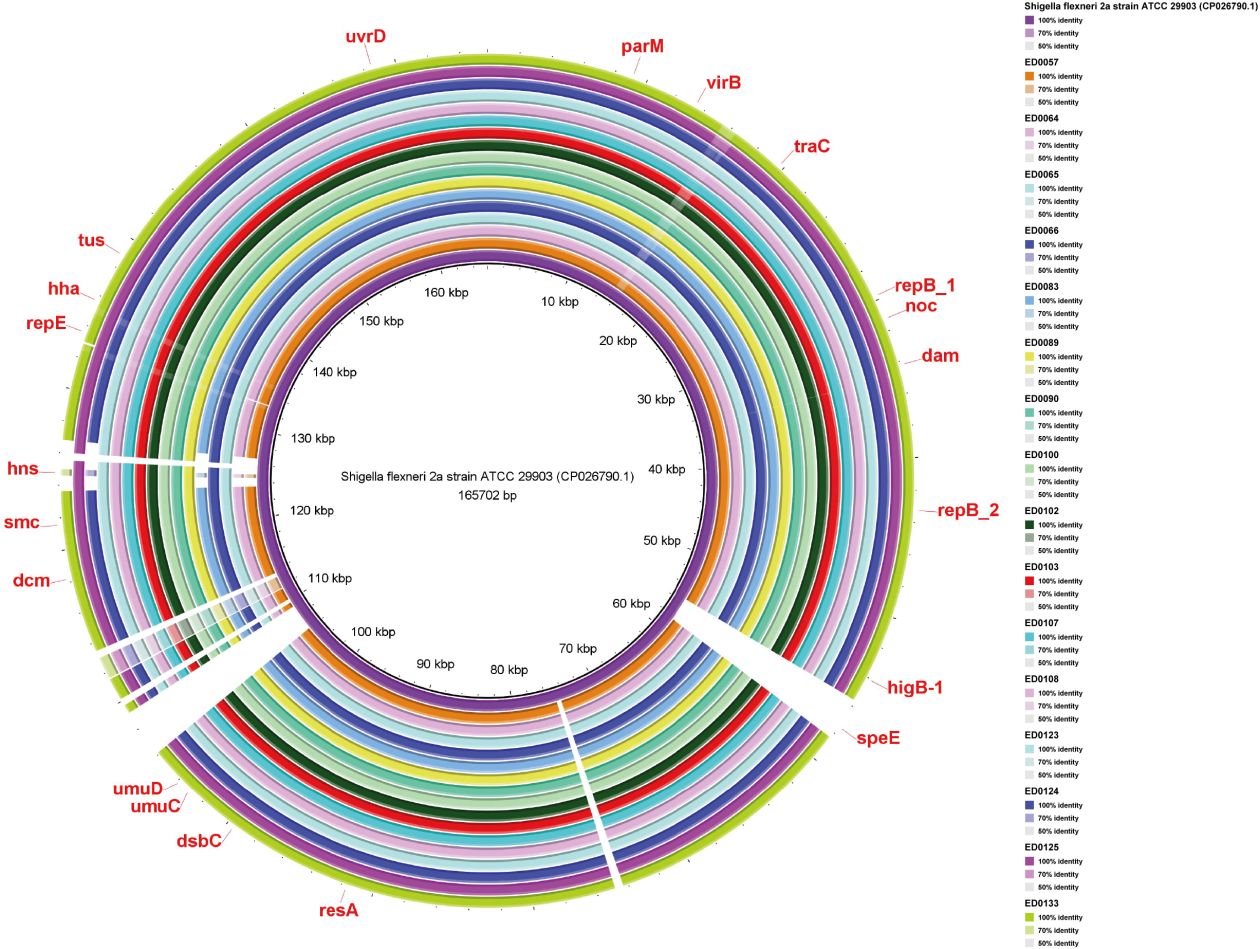
Whole-genome comparison of Blast Ring Image Generated for *Shigella flexneri* 2a (CP026790.1) plasmid in O139:H1 STEC strains isolated from pigs affected by Edema disease in Italy.

*Shigella flexneri* 2a plasmid, harbored *speE*, *higB_1*, *RepB_1*, *RepB_2*, *dam*, *noc*, *traC*, *virB*, *parM*, *uvrD*, *tus*, *hha*, *repE*, *hns*, *smc*, *dcm*, *umuD*, *umuC*, *dsbC*, and *resA* genes (**Figure 5**).

**Figure 6:**
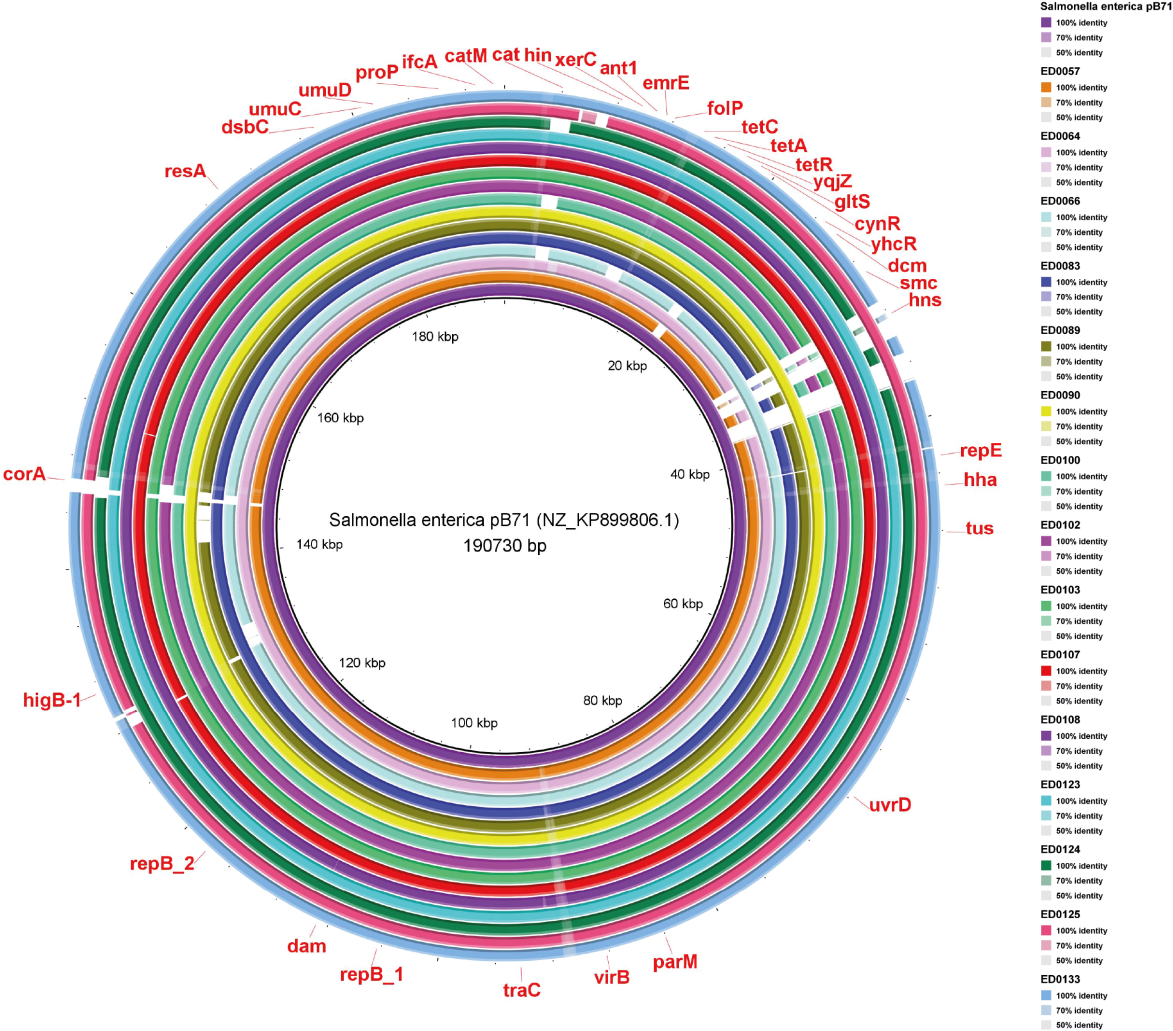
Whole-genome comparison of Blast Ring Image Generated for *Salmonella enterica* pB71 plasmid in O139:H1 STEC strains isolated from pigs affected by Edema disease in Italy.

Ultimately, in pB71 plasmid, the genes *uvrD*, *tus*, *hha*, *repE*, *hns*, *smc*, *dcm*, *yhcR*, *cynR*, *gltS*, *yqjZ*, *tetR*, *tetA*, *tetC*, *folP*, *emrE*, *ant1*, *xerC*, *hin*, *cat*, *catM*, *ifcA*, *proP*, *umuD*, *umuC*, *dsbC*, *resA*, *corA*, *higB-1*, *repB_2*, *dam*, *repB_1*, *traC*, *virB*, and *parM* were present (**Figure 6**). Most of the genes detected were present in all the reference plasmid sequences identified in the studied genomes (**Figures 4**-**6**).

The virulence factors *hlyA*, *hlyB*, *aidA-I*, and *faeE* were identified on pW1316-2 plasmid and *hns*, *traC* and *hha* were detected both on the *Shigella flexneri* 2a and pB71 plasmids according to the VFDB web server (**Table 1**).

**Table 1:** Genomic characterization of the reference plasmids pW1316-2, *Shigella flexneri* 2a, and pB71 in the studied O139:H1 STEC genomes.

Plasmid	Replicon type	Resistance gene	Virulence gene	Length (bp)	Accession number
pW1316-2	IncFII/IncX1	No known genes	*hlyA*, *hlyB*, *aidA-I*, *faeE*	54,694	NZ_CP080237.1
*Shigella flexneri* 2a	IncHI1B(R27)	No known genes	*hns, traC, hha*	165,702	CP026790.1
pB71	IncFIA(HI1)	*tetA, tetC, tetR*	*hns, traC, hha*	190,730	NZ_KP899806.1

Based on the ResFinder analysis on the reference plasmids, the pW1316-2 and *Shigella flexneri* 2a plasmids had no known antibiotic resistance genes; and in the pB71 plasmid, we detected *tet* genes (*tetA, tetC*, and *tetR*) responsible for Tetracycline antibiotic resistance (**Table 1**).

### *Hha-H-NS* complex of *Shigella flexneri* 2a plasmid

**Figure 7:**
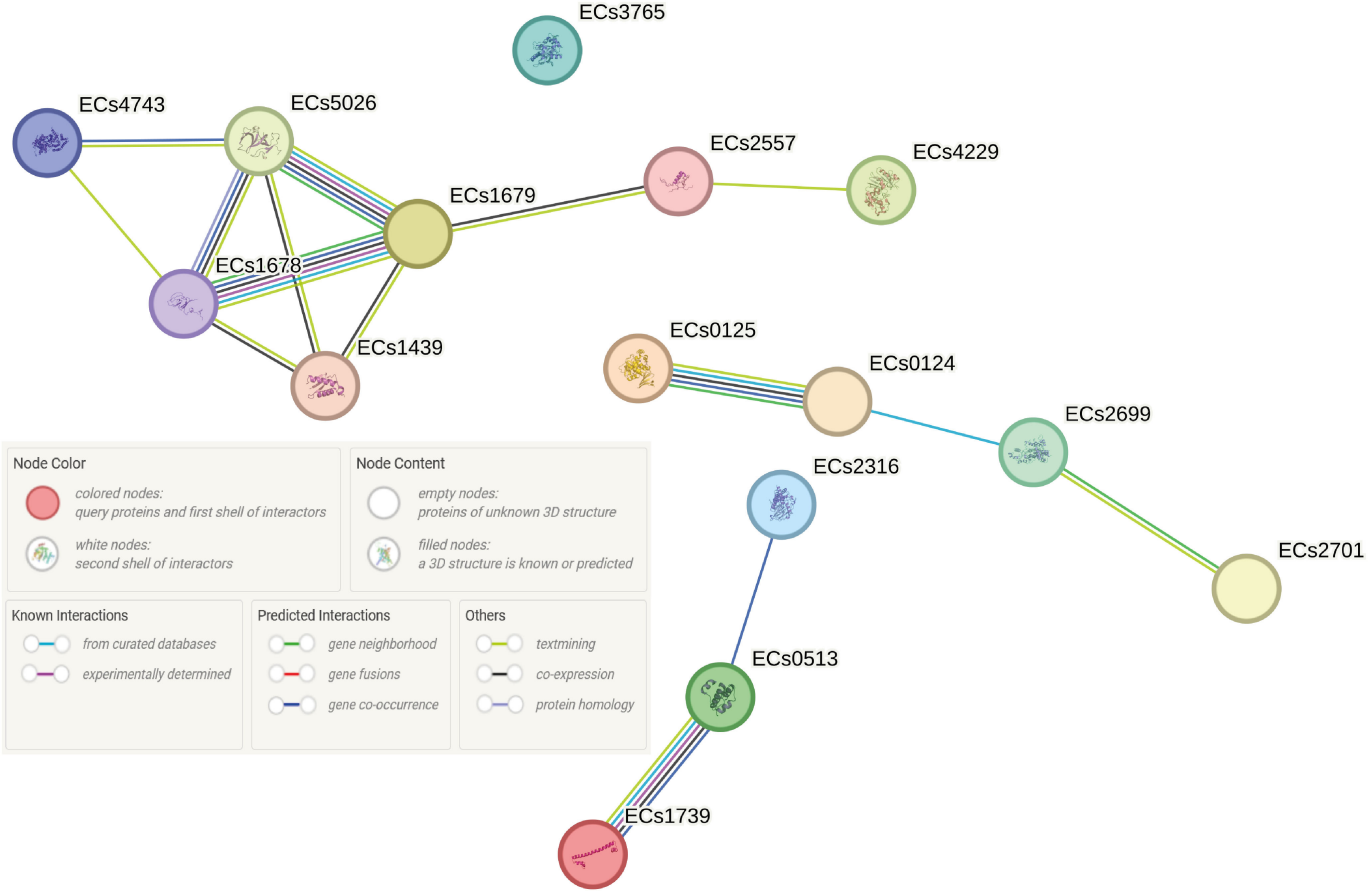
Functional and regulatory associations of genes encoded on the *Shigella flexneri* 2a reference plasmid were examined using the STRING database in O139:H1 STEC strains isolated from pigs affected by Edema disease in Italy. The genes analyzed include ECs1739 (*hns*), ECs0125 (*speE*), ECs1679 (*umuC*), ECs4229 (*dam*), ECs0513 (*hha*), ECs2699 (*dcm*), ECs3765 (*dsbC*), ECs2316 (*tus*), ECs4743 (*uvrD*), and ECs1678 (*umuD*).

Amid the identified plasmids, we observed functional and regulatory associations among the genes encoded on the reference plasmid derived from the *Shigella flexneri* 2a (**Figure 7**). The core of these clustering is centered around the *Hha-H-NS* complex, the functions of which ultimately lead to the production of α-hemolysin, an important virulence factor in ED (Details of associations are provided in tabular form in **Supplementary Table 3**).

### Core genome–based phylogenetic analysis

**Figure 8:**
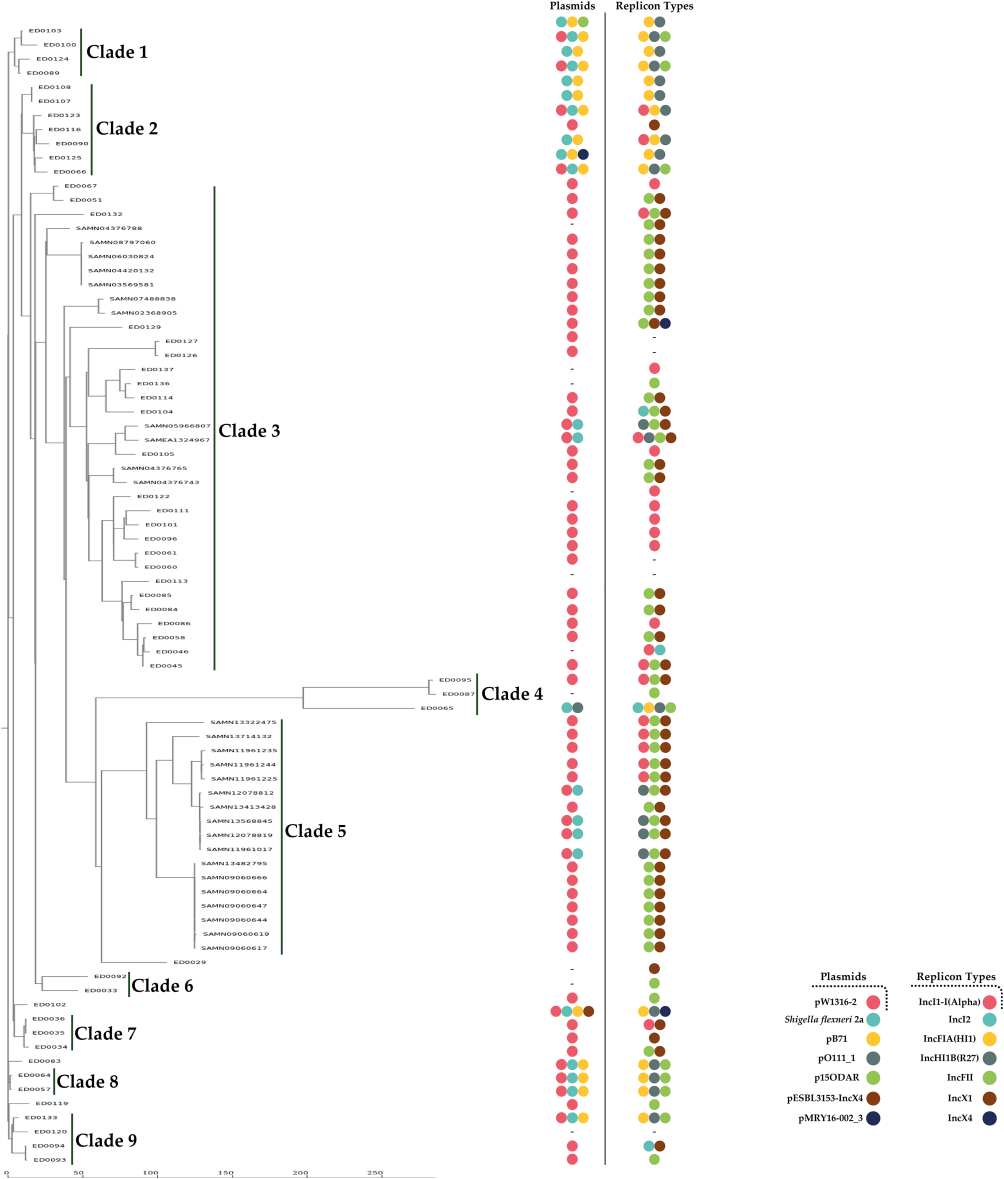
Phylogenetic analysis of O139:H1 STEC strains isolated from pigs affected by Edema disease in Italy. The analysis also included 28 additional O139:H1 STEC genomes from different studies retrieved from public repositories (GenBank and ENA databases). Each entry on the phylogenetic tree shows the strain name, the clade number, the corresponding plasmid, and replicon type, with different colors representing plasmid and replicon types. The scale bar reflects the number of allelic differences.

To explore the phylogenetic relationships and their association with plasmid characteristics, we conducted a cluster analysis using cgMLST for comparative purposes. In addition, we included 28 STEC O139:H1 genomes from various global sources, retrieved from GenBank and ENA databases (**Supplementary Table 2**). We then calculated the number of allelic differences between strains (**Supplementary Table 4**). The analysis grouped the strains into nine main clades (**Figure 8**). Clades 1-2 and 6-9 exhibited significantly lower allelic distances (AD) compared to the other clades, all showing fewer than 90 AD. The majority of the 28 additional strains from external studies were incorporated into clade 5, which was phylogenetically close to clade 3, with most strains showing fewer than 30 AD relative to clade 3 (**Figure 8**). The most genetically distant strains were found in clade 4 (**Figure 8**). Notably, a remarkable correlation was observed between allelic differences in the cgMLST analysis and plasmid characteristics, with strains carrying specific plasmids and replicon types clustering into particular phylogenetic clades (**Figure 8**).

## Discussion

The understanding of ED pathogenesis is crucial due to its significant impact on the swine industry (Imberechts et al., 1992; Gale and Velazquez, 2020). This study undertook a whole genome analysis of O139 STEC isolates collected from pigs with ED to elucidate the role of virulence plasmids on ED development, including adhesion, invasion, and colonization.

The predominance of the reference plasmid pW1316-2, identified through its similarity with genomes, indicates that it is a well-represented plasmid among porcine O139:H1 STEC strains, highlighting its importance in the pathogenesis of ED in pigs. This plasmid, harboring genes encoding hemolysin toxin (*hlyA* and *hlyB*) and adhesins (*aidA-I*, *faeE*), highlights its importance in the pathogenicity (Gale and Velazquez, 2020; Perrat et al., 2022). The presence of *hlyA* and *hlyB*, related with the potent hemolytic activity characteristic of STEC strains associated with ED, emphasizes the cytotoxic potential of pW1316-2 and its implication in the progression of the disease (Holland et al., 1990; Menestrina et al., 1994; Welch, 2005). Furthermore, the involvement of *aidA-I* and *faeE* in adhesion mechanisms crucial for host colonization underscores the importance of pW1316-2 in establishing STEC infections in porcine hosts (Craig, 1906; Niewerth et al., 2001). The assignment of replicon types IncFII/IncX1 to pW1316-2 reference plasmid aligns with previous reports, suggesting the stability and widespread distribution of this plasmid among O139:H1 STEC strains (Nagy et al., 1997; Perrat et al., 2022). Notably, the absence of identified resistance genes on pW1316-2 indicates that this plasmid may not contribute to the dissemination of antibiotic resistance in O139:H1 STEC populations.

The detection of similarity within the genomes to a plasmid present in *Shigella flexneri* 2a among porcine O139:H1 STEC strains indicates intriguing insights into the potential interplay between *Shigella* virulence factors and STEC pathogenesis. This plasmid, harboring genes such as *hns*, *traC*, and *hha*, known for their roles in gene regulation, conjugative transfer, and toxin production, respectively, presents a unique molecular landscape contributing to STEC pathogenesis (Nieto et al., 1991; Schandel et al., 1992; Müller et al., 2006). The association between *hns* gene and the upregulation of key virulence factors, including α-hemolysin and fimbriae, indicates its significance in driving the progression of porcine ED (Müller et al., 2006). Additionally, the presence of *traC* gene, responsible for the synthesis and assembly of the F pilus, highlights the potential for horizontal transfer of this plasmid among O139:H1 strains, potentially contributing to the dissemination of virulence traits via conjugation mediated by F pili (Schandel et al., 1992). Our investigation utilizing the String server has elucidated associations between the *hns* and *hha* genes, indicating their collaborative involvement in regulating the expression of the *hly* operon (Madrid et al., 2002). The *Hha*-*H-NS* complex is important in controlling the production of α-hemolysin, which is crucial in diseases caused by STEC, like ED in pigs (Nieto et al., 2000). The replicon IncHI1B(R27) was found in the *Shigella flexneri* 2a reference plasmid, a type frequently seen in such plasmids (Beloin and Dorman, 2003; Wei et al., 2003). Interestingly, there appears to be a connection between IncHI1B(R27), *hns*, and *hha* genes, as the *Shigella flexneri* 2a plasmid containing the IncHI1B(R27) replicon also houses the *hns* and *hha* genes (Doyle and Dorman, 2006). The absence of antimicrobial resistance genes in the *Shigella flexneri* 2a reference plasmid suggests that, akin to pW1316-2, it may not contribute to the emergence of antibiotic-resistant O139:H1 STEC strains (Beloin and Dorman, 2003; Wei et al., 2003). Moreover, the absence of *IpaH* family genes in the *Shigella flexneri* 2a reference plasmid aligns with the observation that ED-associated *E. coli* strains are typically non-invasive (Tabaran and Tabaran, 2019). Together, these findings reveal the associations and clustering involved in the pathogenicity of porcine O139:H1 STEC strains and emphasize the need for additional research to understand how the *Shigella flexneri* 2a plasmid interacts with STEC virulence.

The identification of similarity to the reference plasmid pB71 among porcine O139:H1 STEC strains reveals a novel dimension in the virulence and resistance profile of these pathogens. Our findings suggest that pB71, which shares virulence genes *hns*, *traC*, and *hha* with plasmid *Shigella flexneri* 2a, may contribute to the pathogenicity of STEC isolates by enhancing the manifestations of ED in pigs (Forns et al., 2005). Moreover, conjugative plasmids within the IncHI1 group play a significant role in disseminating antibiotic resistance among *Salmonella enterica* (Kubasova et al., 2016; Hounmanou et al., 2021). The identification of the IncFIA(HI1) replicon within pB71 suggests its potential involvement in the transmission of antibiotic resistance. This is supported by our finding of tetracycline resistance genes *tetA*, *tetC*, and *tetR* on this plasmid (Bryan et al., 2004; Olowe et al., 2013; Shi et al., 2021). The widespread use of tetracycline in pig farming raises worries about antibiotic resistance in the swine industry due to the rise of pB71-carrying STEC strains resistant to tetracycline (Herrero-Fresno et al., 2017; Abubakar et al., 2019; Græsbøll et al., 2019). Overall, the presence of pB71 reference plasmid underscores the complex interplay between virulence and resistance mechanisms in O139:H1 STEC strains, emphasizing the need for continued surveillance and intervention strategies to mitigate the risk of antimicrobial resistance in swine populations.

Within the various clades identified in the phylogenetic tree from the cgMLST analysis, the pW1316-2 plasmid and the IncFII replicon type were commonly found in the strains, suggesting that this plasmid may have been selected and stabilized within the O139:H1 populations (Perrat et al., 2022). Additionally, the O139:H1 STEC isolates from this study, along with others harboring extra plasmids such as *Shigella flexneri* 2a and pB71, formed a distinct population of STEC strains. With few exceptions, these strains grouped together in clades with small allelic differences and were separate from strains that only had the pW1316-2 plasmid. This suggests that these strains might belong to different lineages, which possibly emerged after the pW1316-2 plasmid spread within the O139 STEC population.

## Conclusions

This study provides new data that augment our understanding of the role of virulence plasmids in the pathogenesis of ED, attributed to porcine O139:H1 STEC strains, showing how important this is for swine industry. The high prevalence of reference plasmid pW1316-2 emphasizes its crucial involvement in causing ED because it carries virulence genes, particularly those related to adhesion mechanisms important for host colonization. The detection of *Shigella flexneri* 2a reference plasmid presents interesting perspectives on possible associations in virulence, while the identification of reference plasmid pB71 reveals a novel dimension in virulence and resistance profiles of O139:H1 STEC strains, raising concerns about antibiotic resistance dissemination in pig farming.

## Data Availability

The datasets presented in this study can be found in online repositories. The names of the repository/repositories and accession number(s) can be found in the article/**Supplementary Material**.
